# The molecular mechanisms of CTHRC1 in gastric cancer by integrating TCGA, GEO and GSA datasets

**DOI:** 10.3389/fgene.2022.900124

**Published:** 2022-07-19

**Authors:** Lulu Zhao, Wanqing Wang, Penghui Niu, Xiaoyi Luan, Dongbing Zhao, Yingtai Chen

**Affiliations:** National Cancer Center/National Clinical Research Center for Cancer/Cancer Hospital, Chinese Academy of Medical Sciences and Peking Union Medical College, Beijing, China

**Keywords:** CTHRC1, gastric cancer, tumor microenvironment, macrophage infiltration, angiogenesis

## Abstract

Collagen triple helix repeat containing-1 (CTHRC1), highly expressed in multiple human solid tumors, has been identified as a tumor associated protein. However, its specific role and mechanism with immune infiltrates in gastric cancer are still unclear. In this study, we systematically explored and validated the expression and prognostic value of CTHRC1 in gastric cancer by integrating the Cancer Genome Atlas (TCGA), Gene Expression Omnibus (GEO) and Genome Sequence Archive (GSA) datasets. Compared to adjacent normal tissues, we observed that CTHRC1 was highly overexpressed in tumor sample of multiple cancers. It was revealed that CTHRC1 overexpression was positively correlated with the T stage in gastric cancer but not lymph nodes metastasis from TCGA dataset. In addition, CTHRC1 expression may induce tumor associated macrophage infiltration though GRN/TNFRSF1A and AnxA1/FPR1 pathways and also tumor angiogenesis in gastric cancer. In this context, our results indicate that CTHRC1 plays a pivotal role in regulating the angiogenesis and macrophage infiltration in tumor microenvironment, and also can predict poor prognosis in gastric cancer, suggesting that CTHRC1 might be a promising novel immunotherapy and angiogenesis target for gastric cancer.

## 1 Introduction

As the fourth leading cause of cancer-related deaths and the fifth most common cancer globally, gastric cancer has poor long-term prognosis, despite of the continuous improvement of effective early screening tools and chemotherapy (Sung et al., 2021). Based on the better understanding of the cellular composition and their crosstalk in tumors in recent years, novel therapeutic targets like PD-L1 (programmed death one ligand-1), PD-1 (programmed cell death protein 1), FGFR2 (fibroblast growth factor receptor 2), and et al., are emerging to prolong the prognosis of gastric cancer patients (Vrána et al., 2018; Xie et al., 2019; Zeng et al., 2019). In this context, an increasing number of specific genomic alterations can potentially serve as treatment targets, and thus assist in reducing the mortality of gastric cancer patients.

As an ECM (extracellular matrix) related protein, Collagen Triple Helix Repeat Containing-1 (CTHRC1) was initially identified in a screening study on differently expressed genes (DEGs) in balloon-injured versus normal rat arteries (Pyagay et al., 2005). Increasing studies have shown that CTHRC1 is aberrantly upregulated in multiple human solid tumors and functionally associated with tumorigenesis, proliferation, invasion, and metastasis (Mei et al., 2020). Specifically, it has been reported that CTHRC1 regulates tumor progression through the CTHRC1/Wnt/β-catenin pathways in breast cancer (Lai et al., 2017), non-small cell lung cancer (Ke et al., 2014), and oral cancer (Liu et al., 2013). Another newly published study showed that the CTHRC1 promotes liver metastasis by reshaping infiltrated macrophages through physical interactions with TGF-βreceptors in colorectal cancer (Zhang et al., 2021). Although CTHRC1 was a promising target for several cancer diseases, its specific role and mechanism with immune infiltrates in gastric cancer are still unclear.

Here, we systematically investigated the expression status and prognostic value of CTHRC1 in gastric cancer by integrating the Cancer Genome Atlas (TCGA), Gene Expression Omnibus (GEO) and Genome Sequence Archive (GSA) datasets. In addition, we further explored the mechanisms of CTHRC1 in the progression of gastric cancer by focusing on the potential function of CTHRC1 in both macrophage infiltration and angiogenesis to identify a novel immunotherapy and angiogenesis target for gastric cancer treatment.

## 2 Methods

### 2.1 Data source and processing

#### 2.1.1 TCGA dataset

A total of 375 gastric cancer cases with gene expression data (HTSeq-FPKM) were collected from TCGA (https://tcga-data.nci.nih.gov/tcga/). Next, Level 3 HTSeq-FPKM data of 375 gastric cancer patients were transformed into transcripts per million (TPM) reads for further analyses. In the TCGA dataset, unknown clinical features or unavailable data were regarded as missing values. These data were divided into two groups, the CTHRC1 high group and the CTHRC1 low group, which accords to the median level CTHRC1 expression.

### 2.2 GEO dataset

A high-throughput platform (GSE84437, Illumina HumanHT-12 V3.0 expression beadchip) was used for the external validation of the CTHRC1 gene in this study. The expression values at the probe level (probe ID) were converted to the corresponding gene symbol according to their annotation files without further standardization. If multiple probes matched with the same gene, the average value was calculated as the expression value of the gene. Gene expression data together with corresponding clinical information were downloaded from GEO dataset (https://www.ncbi.nlm.nih.gov/geo/). Finally, totally 433 patients with gastric cancer were enrolled for analysis.

### 2.3 Single-cell RNA sequencing (scRNA-seq) data from GSA

In our previous study, we performed the scRNA-seq on nine untreated non-metastatic gastric cancer patients (Li et al., 2022). A total of 47,304 cells with detectable expression of more than 200 genes were obtained after quality control. Here, in order to explore the CTHRC1 gene, we downloaded our previous scRNA-seq data in this study for more analysis (https://ngdc.cncb.ac.cn/gsa/, data number: CRA002586). Canonical correlation analysis (CCA) (Butler et al., 2018) in Seurat was first applied to the combined gene expression matrix of all samples to correct the batch effects among the experiments. Then, the CCA object was aligned with the *Align Subspace* function to return a new dimensional reduction that was used in cell clustering. Principle component analysis (PCA) was conducted to reduce the dimensionality of the expression matrix. T-SNE dimensionality reduction was performed on the first 30 principle components using the *FindCluster* function in the Seurat package with the default parameters. Canonical marker genes were used to identify the resulting cell clusters as known cell types.

### 2.4 Functional enrichment analysis

To elucidate the potential functions of CTHRC1, we analyzed the DEGs between the CTHRC1 high and CTHRC1 low groups with R package DESeq2 (Love et al., 2014), by setting a log-fold change larger than 1.5 and an adjusted *p* value less than 0.05. The identified DEGs were then processed for functional annotation on the Metascape database2 (http://metascape.org) (Zhou et al., 2019). Minimum counts larger than 3, enrichment factors larger than 1.5, and a *p* value less than 0.01 were set as analysis thresholds.

Gene set enrichment analysis (GSEA) (https://www.gsea-msigdb.org) as used to conduct a gene set enrichment analysis and determine whether a defined set of genes exhibits statistically significant concordant differences between two biological states. In this study, GSEA was performed to elucidate the significant function and pathway differences between the high and low CTHRC1 expression groups using R package clusterProfiler (Yu et al., 2012). A pathway term with normalized enrichment score (NES) > 1, adjusted *p* value < 0.05, and false discovery rate (FDR) < 0.25 was considered to be significantly enriched.

### 2.5 TIMER database analysis

TIMER database (https://cistrome.shinyapps.io/timer/) is a comprehensive web platform, which contains 10,897 samples for the systematic analyses of immune infiltrates across many cancers from the TCGA dataset (Li et al., 2017). The gene module was used to visualize the correlation of CTHRC1 expression with immune infiltration level in gastric cancer.

### 2.6 Cell-cell interaction analysis

We used CellPhoneDB (v.1.1.0) to detect the pairwise interactions between cell clusters (Efremova et al., 2020). Only receptors and ligands whose expression was detected in more than 25% of cells were included in this analysis. The significance of a ligand and receptor pair in each cell-cell interaction was evaluated by 1,000 random permutations of the cell types. For each permutation, the total mean of the average receptor expression level and the average ligand expression level is calculated, and a null distribution is derived for each ligand-receptor pair.

### 2.7 Immunofluorescence staining

To confirm the association of CTHRC1 and blood vessel in gastric tumors, we performed immunofluorescence staining of CTHRC1 in the tumor samples. Serial sections (about 4 μm) were obtained from the formalin-fixed paraffin-embedded tumor tissues and stained by use of standard protocols. Anti-CD31 (mouse, 1:50, Proteintech, Ag1787, lot No. 66065-1-Ig) was used to stain endothelial cells, and the corresponding proteins were detected with anti-CTHRC1 (rabbit, 1:50, Proteintech, Ag9812, lot No. 16534-1-AP) and dilutions. DAPI was used to stain cell nuclei.

### 2.8 Statistical analyses

Spearman correlation analyses were used to assess the correlations between continuous variables. Differences in the variables between the CTHRC1 high and CTHRC1 low groups were evaluated with the Student t test, one-way ANOVA, or Pearson’s chi-squared test. The survival data was obtained from TCGA and GSE84437 datasets, while Kaplan-Meier method and Cox proportional hazards regression analysis were used to evaluate prognostic factors. Results with a *p* value < 0.05 were considered significant. All statistical analyses were performed using R v3.6.1 (https://www.r-project.org/) and Prism 8.0.1 (GraphPad Software Inc.).

## 3 Results

### 3.1 The expression level of CTHRC1 in gastric cancer

**FIGURE 1:**
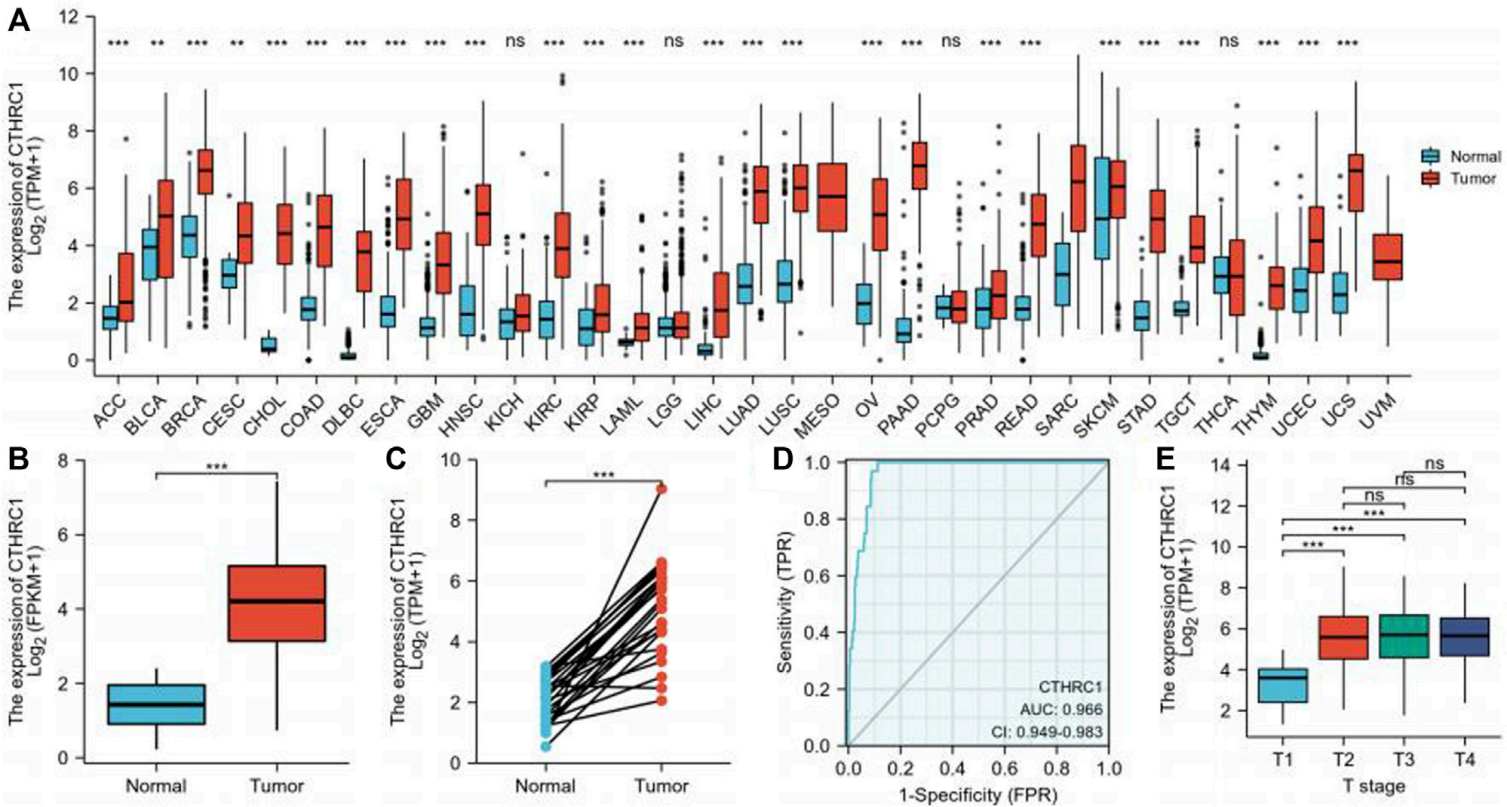
CTHRC1 Expression Levels in gastric cancer. **(A)** Higher or lower CTHRC1 expression in multiple tumors when compared with normal tissues across TCGA and the Genotype-Tissue Expression (GTEx). **(B)** CTHRC1 expression levels in gastric cancer and adjacent normal tissues across TCGA. **(C)** The expression of CTHRC1 in gastric cancer and its paired adjacent tissues. **(D)** Operating characteristic analysis (ROC) of CTHRC1 in gastric cancer. **(E)** CTHRC1 expression pattern by different T stage of gastric cancer. (*p < 0.05, **p < 0.01, ***p < 0.001).

The pan-cancer analyses were performed to compare the expression of CTHRC1 in the tumor samples of TCGA combined with normal samples of TCGA and the Genotype-Tissue Expression (GTEx) by Wilcoxon rank sum test (Supplementary Table S1). We found that CTHRC1 gene was highly expressed in 26 types of tumor (*p* < 0.05) (Figure 1A). Figure 1B showed the comparison of the CTHRC1 gene in 32 gastric cancer samples and 375 GC samples from TCGA dataset (*p* < 0.001). Furthermore, we compared the expression of CTHRC1 in 27 gastric cancer samples and matched normal samples (*p <* 0.01, Figure 1C).

Receiver operating characteristic curve (ROC) was used to analyze the distinguishing efficacy of CTHRC1 between tumor tissues and normal tissue (Figure 1D). The area under the curve (AUC) of CTHRC1 is 0.966, suggesting that CTHRC1 shows significant high sensitivity and specificity for the diagnosis of gastric cancer. To better understand the relevance and underlying mechanisms of CTHRC1 expression, we investigated the relationship between the CTHRC1 expression and clinical characteristics of gastric cancer samples. As shown in Table 1 and Figure 1E, increased CTHRC1 expression was enriched in T4 stage (*p* < 0.01).

**TABLE 1:** CTHRC1 expression and clinical characteristics in gastric cancer.

Characteristic		CTHRC1 low (*n* = 187)	CTHRC1 high (*n* = 188)	*p* value
Age	≤65	83 (22.4%)	81 (21.8%)	0.737
	>65	100 (27%)	107 (28.8%)	
Age, meidan (IQR)	67 (58, 73)	68 (59, 74)	0.641	
Gender	Female	68 (18.1%)	66 (17.6%)	0.884
	Male	119 (31.7%)	122 (32.5%)	
*H pylori* infection	No	97 (59.5%)	48 (29.4%)	0.822
	Yes	11 (6.7%)	7 (4.3%)	
Histologic grade	G1	5 (1.4%)	5 (1.4%)	0.123
	G2	77 (21%)	60 (16.4%)	
	G3	99 (27%)	120 (32.8%)	
T stage	T1	19 (5.2%)	0 (0%)	<0.001
	T2	40 (10.9%)	40 (10.9%)	
	T3	80 (21.8%)	88 (24%)	
	T4	48 (13.1%)	52 (14.2%)	
N stage	N0	51 (14.3%)	60 (16.8%)	0.718
	N1	50 (14%)	47 (13.2%)	
	N2	40 (11.2%)	35 (9.8%)	
	N3	39 (10.9%)	35 (9.8%)	
M stage	M0	165 (46.5%)	165 (46.5%)	1
	M1	12 (3.4%)	13 (3.7%)	
Residual tumor	R0	154 (46.8%)	144 (43.8%)	0.933
	R1	8 (2.4%)	7 (2.1%)	
	R2	9 (2.7%)	7 (2.1%)	

### 3.2 Univariate and multivariate analysis of survival outcomes

**FIGURE 2:**
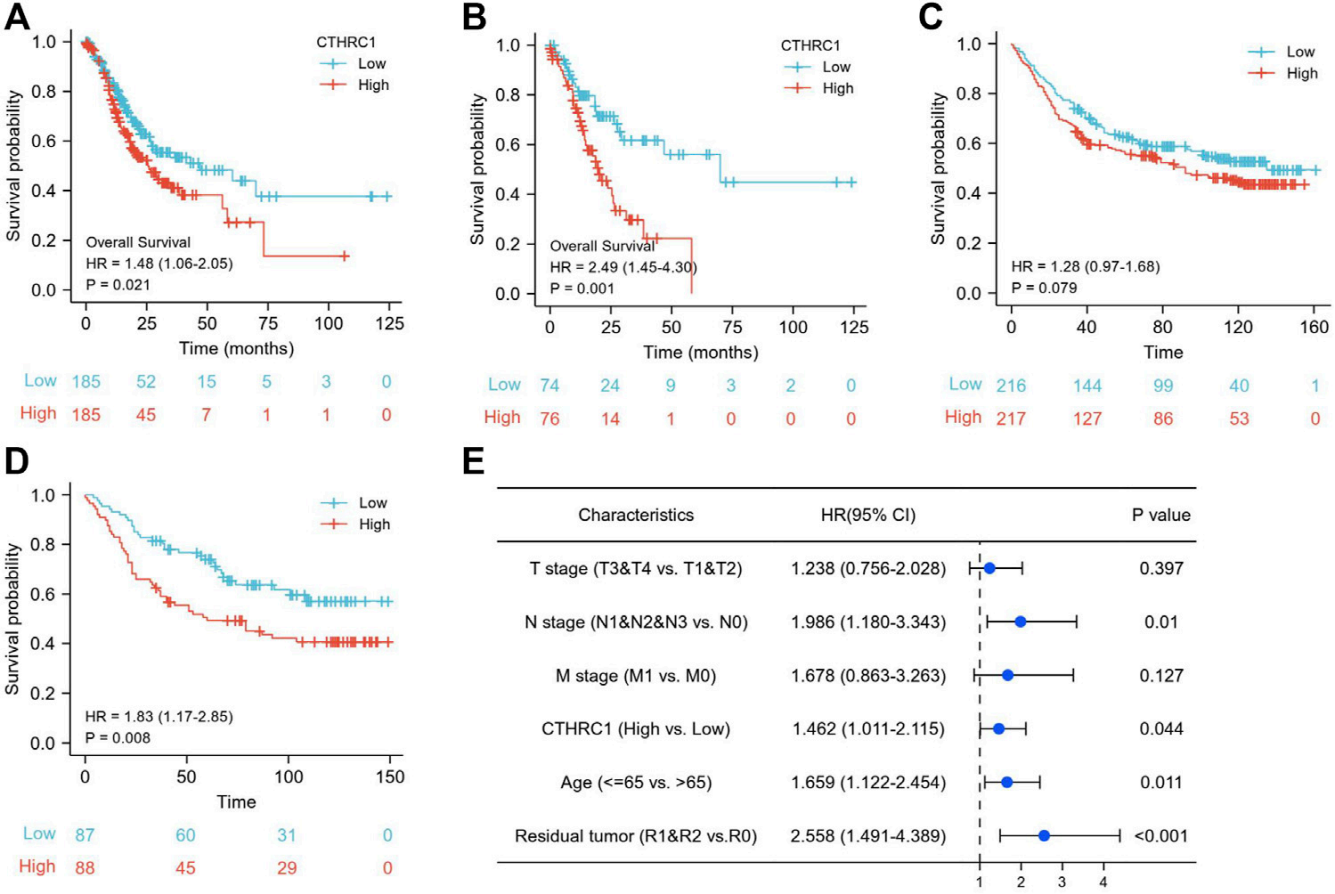
The prognostic value of CTHRC1 expression in gastric cancer. **(A)** Kaplan-Meier survival curves comparing the high and low expression of CTHRC1 in gastric cancer using TCGA dataset, and **(B)** also the outcomes with 20% cut off value. **(C)** Kaplan-Meier survival curves comparing the high and low expression of CTHRC1 in gastric cancer using GSE84437 dataset, and **(D)** also the outcomes with 20% cut off value. **(E)** The survival of multivariate analysis using TCGA dataset.

According to the Kaplan-Meier survival analysis, the low CTHRC1 group (n = 187) showed a better prognosis than the high CTHRC1 group (*n* = 188) (Figure 2A, HR = 1.48, 95%CI: 1.06–2.05, *p* = 0.021). We further selected the top 20% patients (*n* = 75) with high or low CTHRC1 expression form TCGA dataset to analyses (Figure 2B), which showed similar OS results (HR = 2.49, 95CI:1.45–4.30 *p* = 0.021). To validate the association between CTHRC1 expression and survival outcome, we also used the GSE84437 dataset (*n* = 433) from the GEO database to verify it. The result demonstrated the same survival trend in both 50% cut-off value (216 vs. 217) and 20% cut-off value (43 vs. 44) groups (Figures 2C, D, *p* = 0.079 and *p* = 0.008, respectively).

Figure 2E showed the multivariate analysis after adjusting T stage, N stage, M stage, age and residual tumor, which produced an HR of 1.462 (95% CI: 1.011–2.115, *p* = 0.044), suggesting that CTHRC1 gene was independently associated with mortality of gastric cancer patients.

### 3.3 CTHRC1 related biological process

**FIGURE 3:**
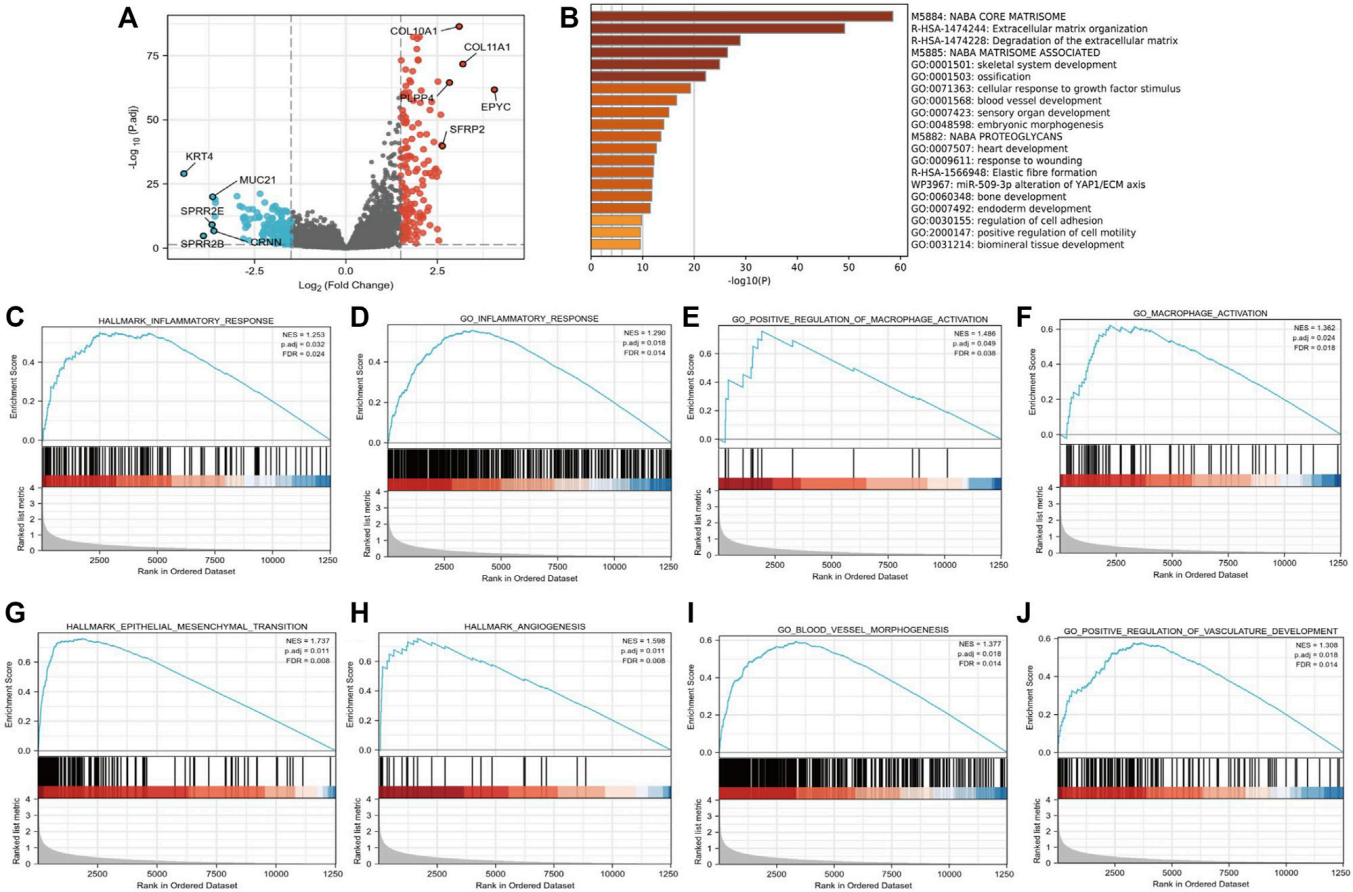
Functional annotation of differentially expressed genes (DEGs) in gastric cancer patients with distinct CTHRC1 levels. **(A)** Volcano plots of the DEGs with the top 10 DEGs. **(B)** Pathways enriched in CTHRC1 high groups using Metascape database. **(C–J)** Representative Gene Set Enrichment Analysis between CTHRC1 high and CTHRC1 low expression groups.

We further compared 188 gastric cancer CTHRC1 high samples with 187 CTHRC1 low controls in Table 1. A total of 297 DEGs, covering 145 upregulated genes and 152 downregulated genes, were identified to be statistically significant between the two cohorts (Fugure 3A; Supplementary Table S2). Relative expression values of the top 10 DEGs between the two cohorts were also showed in Figure 3A.

The software “Metascape” was adopted in order to evaluate the function of CTHRC1-associated DEGs in gastric cancer patients. We found that several pathways were enriched, including extracellular matrix organization and vascular development (Figure 3B). Further, GSEA was conducted between the high CTHRC1 and low CTHRC1 expression datasets to reveal significant differences in enrichment of MSigDB Collection (c2. cp.biocarta and hall. V6.1 symbols). The most significantly enriched signaling pathways based on their NES were selected. Moreover, the differentially enriched pathways in CTHRC1 high expression phenotype include pro-tumor and immune associated pathways (Figures 3C–J), such as hallmark epithelial mesenchymal transition (NES = 1.737, *p* = 0.011, FDR = 0.008), hallmark angiogenesis (NES = 1.598, *p* = 0.011, FDR = 0.008), go blood vessel morphogenesis (NES = 1.377, *p* = 0.018, FDR = 0.014), go positive of vasculature development (NES = 1.308, *p* = 0.018, FDR = 0.014), hallmark inflammatory response (NES = 1.253, *p* = 0.032, FDR = 0.024), go inflammatory response (NES = 1.290, *p* = 0.018, FDR = 0.014), go macrophage activation (NES = 1.362, *p* = 0.024, FDR = 0.018), and go positive regulation of macrophage activation (NES = 1.486, *p* = 0.049, FDR = 0.038).

### 3.4 CTHRC1 expression and macrophages infiltration

**FIGURE 4:**
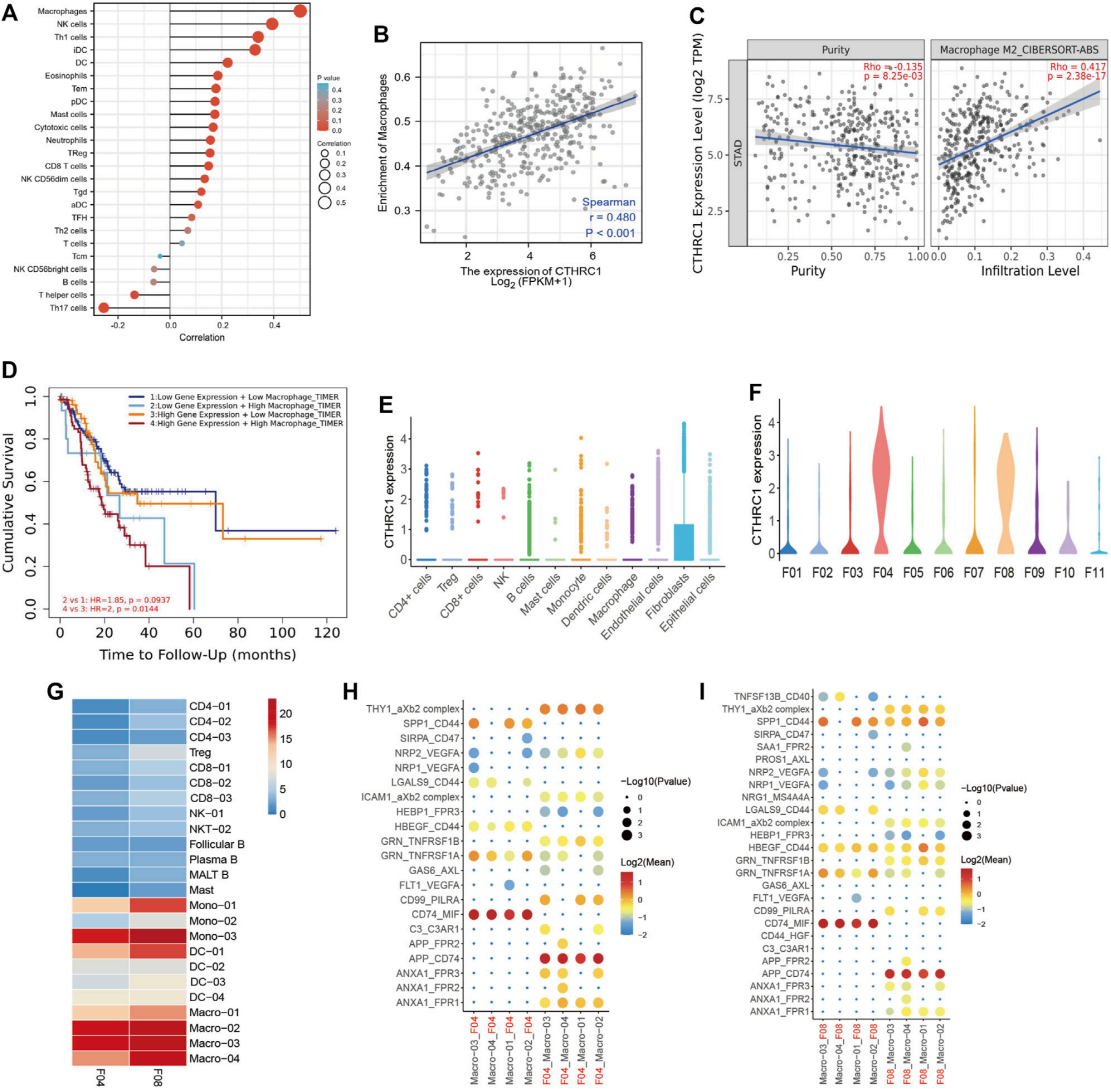
CTHRC1 expression and macrophages infiltration. **(A)** The expression level of CTHRC1 was associated with the immune infiltration in gastric cancer. **(B)** CTHRC1 expression significantly positively correlated with the macrophage infiltration, and **(C)** M2 macrophage infiltration. **(D)** Kaplan-Meier survival curves comparing the high and low expression of macrophages infiltration and CTHRC1 expression. **(E)** The CTHRC1 expression in different cell types in gastric cancer using single cell RNA sequencing. **(F)** The CTHRC1 expression in different fibroblast clusters in gastric cancer using single cell RNA sequencing. **(G)** The cell interaction between tumor cells from F04 and F08 and immune cells. **(H–I)** The ligand-receptor pairs between tumor cells from F04 and F08 and immune cells.

We next analyzed the correlation between the expression level of CTHRC1 and immune cell infiltration level quantified. As showed in Figure 4A, the expression of CTHRC1 was negatively correlated with the abundance of acquired immunocytes, such as helper T17 cell (Th17) and T helper cell, and meanwhile positively correlated with the abundance of innate immunocytes, such as macrophages, natural killer cell (NK), helper T1 cell (Th1), Dendritic Cells (DC), and DC interdigitating (iDC). Furthermore, the analyses showed that the CTHRC1 expression was positively correlated with the infiltration of macrophages, as well as M2 macrophages (R = 0.480, *p* < 0.001, Figures 4B, C).

Thus, we used TIMER software to explore the patients with different level of macrophages infiltration and CTHRC1 expression (Figure 4D). In the high CTHRC1 group, the patients with high macrophages infiltration had worse prognosis than those with low macrophages infiltration (*p* = 0.0144). This tendency was also showed in the low CTHRC1 group, but there was no statistical difference in this aspect between the two groups (*p* = 0.0937).

### 3.5 Validation of CTHRC1 expression in scRNA-seq data

To verify the correlation between CTHRC1 and macrophages, we downloaded our previous scRNA-seq data from GSA for analysis. After identifying major cell types, we found 11 fibroblast clusters (F01-F11) from gastric cancer patients (Li et al., 2022). In addition, F04 and F08 were significantly enriched in tumor samples, thus were denoted as three subsets of cancer associated fibroblasts (CAFs).

In this study, we found that CTHRC1 was mainly expressed in fibroblast cells, especially in F04 and F08 clusters (Figures 4E, F). This may indicate that CTHRC1 highly expressed in CAFs. We next performed predictive analyses of the cell-cell interactions based on the expression of ligand-receptor pairs using the statistical inference framework of CellPhoneDB (Efremova et al., 2020). Briefly, we observed more intensive cell-cell interactions in the macrophages than in the other immune cell types (Figure 4G), which was consistent with the results from TCGA dataset showed above. Furthermore, the interactions of CTHRC1 associated fibroblast cells (F04 and F08) and macrophages were mediated by a different set of ligand-receptor pairs (Figures 4H, I), including THY1_aXb2 complex, GRN_TNFRSF1A, CD99_PILRA, CD74_MIF, APP_CD74, ANXA1_FPR1, etc.

### 3.6 CTHRC1 expression and angiogenesis

**FIGURE 5:**
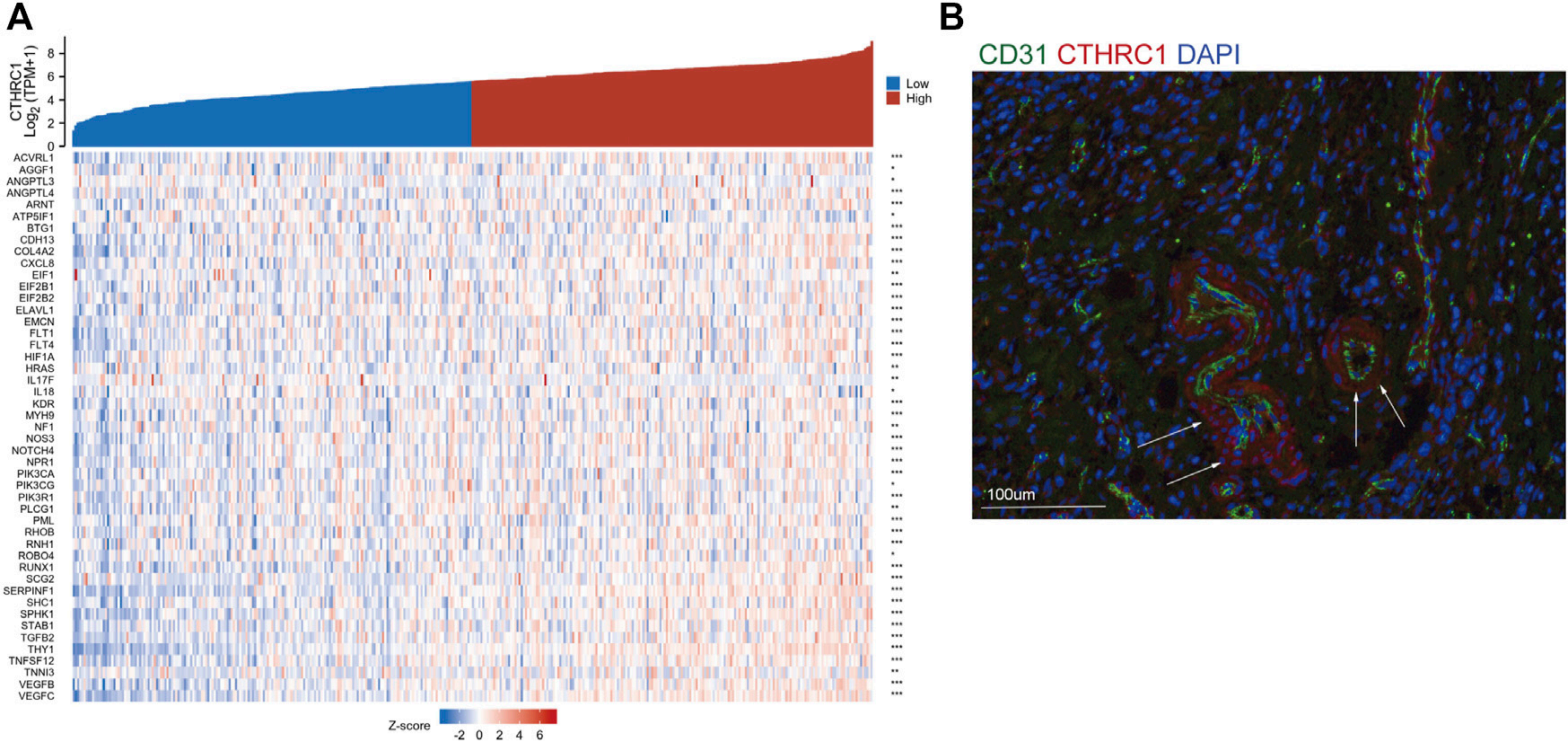
CTHRC1 expression and angiogenesis. **(A)** The co-expression between CTHRC1 and some angiogenesis associated genes. **(B)** Immunofluorescence staining of CTHRC1, CD31 and DAPI.

To verify the correlation between CTHRC1 and angiogenesis, we constructed co-expression analysis between CTHRC1 and some angiogenesis associated markers in gastric cancer using the TCGA dataset. We found many angiogenesis associated genes showed consistent expression trend of CTHRC1 in gastric cancer, such as ACVRL1, ANGPTL3, ANGPTL4, NOTCH4, VEGFB, VEGFC etc. (Figure 5A) Immunofluorescence staining showed that CTHRC1 was present in the vascular tissue surrounding the gastric tumors (Figure 5B), suggesting that CTHRC1 may participate in extracellular matrix remodeling in gastric tumor angiogenesis.

## 4 Discussion

In this study, we presented a comprehensive catalog of CTHRC1 together with its prognostic value and related biological processes in gastric cancer to systematically investigate the expression pattern. We identified that CTHRC1 expression tends to be upregulated in gastric cancer at its later T stage, however, no correlation was found between CTHRC1 expression and lymph node metastasis. In addition, the CTHRC1 high group was associated with worse prognosis in gastric cancer patients in both univariate and multivariate analysis. We also found that CTHRC1 expression was significantly associated with immune response, as well as tumor progression associated pathways, like epithelial mesenchymal transformation and angiogenesis, indicating CTHRC1 gene multi-dimensionally participates the biological process of tumor microenvironment in gastric cancer.

The primary finding of our study was that higher CTHRC1 expression was significantly correlated with macrophages infiltration, although other innate immune cells, like NK cells, Th1 cells and DC cells showed the same trend. We further focus on the M2 like macrophages, and we found M2 macrophages infiltration were strongly associated with CTHRC1 high expression, which provides profound insight into mechanisms governing tumor macrophages infiltration and functional activation. As we all known, M2 macrophages can express a large number of scavenger receptors (Locati et al., 2020), which is related to the high-intensity expression of IL-10, IL-1β and matrix metalloproteins (MMPs) in the tumor microenvironment (Chen et al., 2017), so they have the function of promoting angiogenesis, tissue reconstruction, tumorigenesis and also tumor development.

In order to explore the mechanism of CTHRC1 in inducing macrophage infiltration, we used the scRNA-seq for cell-cell interaction analysis. We found CAFs which highly expressed CTHRC1 (from F04 and F08) and tumor associated macrophages were mediated by a different set of ligand-receptor pairs, including THY1/aXb2 complex, GRN/TNFRSF1A, CD99/PILRA, CD74/MIF, APP/CD74, ANXA1/FPR1, etc.

In our previous study (Li et al., 2022), F04 and F08 were significantly enriched in tumor samples, thus were denoted as three subsets of CAFs. Tang et al. had indicated that GRN bound directly to tumor necrosis factor receptors (TNFRs) and disturbed the TNFα-TNFR interaction (Tang et al., 2011), while TNFα is responsible for a diverse range of signaling events within cells, leading to necrosis or apoptosis, and also important for resistance to cancers (So and Ishii, 2019). In addition, Vecchi has reported that inhibiting AnxA1/FPR1 autocrine axis can reduce breast cancer cell growth and aggressiveness both *in vitro* and *in vivo* (Vecchi et al., 2018). Thus, we may conclude that CTHRC1 may induce tumor associated macrophage infiltration though GRN/TNFRSF1A and AnxA1/FPR1 pathways.

Another concern is the mechanism of CTHRC1 in tumor angiogenesis. Previous studies have demonstrated that CTHRC1 activates HIF-α pathway and contributes to tumor angiogenesis in hepatocellular carcinoma and pancreatic cancer (Zhang et al., 2015; Lee et al., 2016). In gastric cancer, we found CTHRC1 was highly co-expressed with many factors, such as ACVRL1, ANGPTL3, ANGPTL4, NOTCH4, VEGFB, VEGFC etc., which have been proved to play an important role in angiogenesis in various cancer (Siamakpour-Reihani et al., 2015; Zheng et al., 2021). Immunofluorescence staining showed that CTHRC1 was present in the vascular tissue surrounding the gastric tumors, suggesting the high interaction for CTHRC1 and blood vascular development. Furthermore, stromal cells and cancer cells within the tumor microenvironment can be depicted as a highly complex signaling network to influence each other’s function and impact on cancer progression and metastasis. Thus, we guess that tumor-associated macrophages induced by CTHRC1 also serve as angiogenesis promoting cells via the production of pro-angiogenic factors and MMPs and also vascular construction which guarantee the supply of oxygen and nutrients to solid tumor cells (Fu et al., 2020; Qian and Pollard, 2010). The angiogenesis progress is complex in gastric cancer, where inhibiting CTHRC1 may decrease tumor blood vascular development. Future studies are needed to focus on investigating the role of CTHRC1 in regulating the angiogenesis targeted animal models.

This study has several limitations. Firstly, our research does not include the verification of CTHRC1 gene *in vitro* and *in vivo* experiments, which would be our further work. Secondly, the sample size of these datasets, included TCGA, GEO and GSA datasets, is limited. Even though, the results of the present study reveal the molecular mechanisms of CTHRC1 in gastric cancer.

In conclusion, CTHRC1 expression may induce tumor associated macrophage infiltration though GRN/TNFRSF1A and AnxA1/FPR1 pathways and also tumor angiogenesis in gastric cancer. Therefore, our results indicate that CTHRC1 plays a pivotal role in regulating the tumor microenvironment and predicts poor prognosis in gastric cancer, which suggests that CTHRC1 might be a promising novel immunotherapy and angiogenesis target for gastric cancer.

## Data Availability

The original contributions presented in the study are included in the article/Supplementary Material, further inquiries can be directed to the corresponding author.
